# Photobiomodulation versus corticosteroid in the management of erosive oral lichen planus: a randomized controlled clinical trial

**DOI:** 10.1186/s12903-024-03976-6

**Published:** 2024-02-17

**Authors:** Reem Kamal Mohamed, Naguiba Mahmoud Elsayed, Sabah Abdelhady Mahmoud, Yasmine Youssri Gaweesh

**Affiliations:** 1 Department of Oral Diagnosis, Oral Medicine, Periodontology and Oral Radiology, Faculty of Dentistry, Alexandria University, Champollion St., Azarita, Alexandria Governorate, 21527 Egypt; 2 Department of Biochemistry, Faculty of Medicine, Alexandria University, Champollion street،, Al Mesallah Sharq, Al Attarin, Alexandria, Alexandria Governorate, 21568 Egypt

**Keywords:** Oral lichen planus, Photobiomodulation, Topical steroid, Diode laser, Salivary malondialdehyde

## Abstract

**Background:**

Oral lichen planus (OLP) is a chronic illness of immune origin that is typically treated with corticosteroids as a gold standard therapy. Photobiomodulation (PBM) may represent an alternative remedy that has the potential to treat a variety of pathological conditions by alleviating pain, reducing inflammation, and promoting tissue healing without the drawbacks of steroid therapies. Thus, the aim of the current study was to compare the effect of photobiomodulation to topical 0.1% triamcinolone acetonide on erosive oral lichen planus.

**Methods:**

This randomized controlled clinical trial involved 44 patients complaining of erosive oral lichen planus. Patients were assigned to one of two groups: control group (*n* = 22) received 0.1% topical triamcinolone acetonide three times daily with miconazole oral gel once daily for 4 weeks, and photobiomodulation group (*n* = 22) received laser therapy by 980 nm diode laser utilizing output power 300 mW twice weekly for 5 weeks (a total of 10 sessions). The evaluation of patients was performed at baseline, 6 weeks, and 12 weeks postoperatively in terms of pain, clinical scores, and biochemical evaluation of salivary malondialdehyde levels. All recorded data were analyzed using Mann–Whitney test to compare the two studied groups regarding pain, lesion size, and salivary levels of malondialdehyde. Friedman test, followed by post hoc test, was used for comparison of the data within the same group along the 3 periods at baseline, 6 weeks, and 12 weeks.

**Results:**

Both groups showed significant improvement in pain and clinical scores, with no statistical difference between them. Moreover, there was a significant improvement in salivary malondialdehyde levels for both groups, with no significant difference between them.

**Conclusions:**

Photobiomodulation could be a promising therapeutic modality for management of erosive oral lichen planus without the side effects of steroid therapy. The salivary malondialdehyde level could be used as a biomarker to evaluate the disease severity and its response to the treatment.

**Trial registration:**

The study has been registered at ClinicalTrials.gov (NCT05951361) (19/07/2023).

## Background

Oral lichen planus (OLP) is an inflammatory mucocutaneous disease of chronic nature that affects 1%–2% of middle-aged individuals, with a distinct female prevalence [1, 2]. It typically manifests as bilateral, symmetrical lesions, mostly affecting the buccal mucosa, gingiva, dorsum, and margins of the tongue [3].

OLP diagnosis is basically dependent on the characteristic clinical appearance. However, histological findings such as basal keratinocyte death, basement membrane breakdown, and a subepithelial band of lymphocytes exhibiting significant inflammatory infiltration aid in confirming the diagnosis [4].

OLP has different clinical patterns such as reticular, plaque-like, atrophic, erosive/ulcerative, papular, and bullous. Any of these patterns can clinically occur separately or in combination. However, the reticular OLP is the most common one. It is commonly asymptomatic and clinically distinguished by interwoven striae, known as Wickham’s striae in addition to hyperkeratotic plaques and papules [5].

On the other hand, erosive and ulcerated lesions are symptomatic and may cause various levels of pain and discomfort to the patients. As a result, food intake impairment may occur, which could negatively impact their quality of life. Moreover, an increased malignant transformation risk has been associated with several forms of OLP [6].

Therefore, it is essential to manage symptomatic OLP lesions, including erosive, atrophic, and ulcerative lesions through appropriate treatment in order to reduce pain and improve the patient’s well-being [7].

The development of OLP and its potential risk for malignant transformation have been linked to a condition of oxidative stress (OS) [8, 9]. The oxidative stress state contributes to the distinct histopathological features of OLP by facilitating the apoptosis of basal keratinocytes as well as recruitment of T lymphocytes and other inflammatory cells to the OLP lesions [10].

In an oxidative stress state, the capability of reactive oxygen species (ROS) to sustain an inflammatory state in OLP lesions may be linked to their capacity to enhance the expression of pro-inflammatory cytokines (such as tumor necrosis factor α [TNF-α]), which are involved in recruiting T lymphocytes, the activation of matrix metalloproteinase (MMP) enzymes that degenerate the basement membrane, and the modification of intracellular signaling molecules which regulate apoptosis and disintegrate the keratinocytes’ lipid membrane, resulting in further localized tissue damage and excessive ROS production, creating a detrimental cycle [8–11].

Hence, numerous treatment modalities have been investigated in clinical trials in order to manage OLP and reduce the high ROS levels reported in OLP lesions. Moreover, these modalities aim to eliminate corticosteroids’ side effects, which are considered the gold standard therapy for OLP [12, 13].

The non-invasive, non-ablative technique of photobiomodulation (PBM) has emerged as a remedy for OLP owing to its capacity to minimize pain, eliminate inflammation, and encourage tissue regeneration [14].

PBM has numerous impacts at the molecular, cellular, and tissue levels. There is substantial proof that it influences the mitochondria inside the cell to boost the production of adenosine triphosphate (ATP), control ROS, and manage the transcription factors responsible for protein synthesis. Additionally, it modifies cytokine levels, growth hormones, lowers oxidative stress, and improves oxygen supply to the tissues [15, 16].

The mechanism of PBM on cellular level was explained by Hamblin (2018), who stated that, the use of wavelengths close to infrared may impact molecules that absorb light, such as cytochrome c oxidase (CCCO), resulting in changes in the activity of mitochondria via a redox reaction breaking the bond between cytochrome c oxidase (CCCO) and nitric oxide (NO), which leads to the activated CCCO resulting in an increase in the production and release of ATP, a reduction of reactive oxygen species (ROS), as well as the activation of RNA transcription and the synthesis of DNA, a process that benefits the repair and healing of cells. Indirect effects such as the release of nitric oxide through the activity of the electron transport chain, the process that enhances the dilation of local blood vessels, the availability of oxygen, and the permeability of cells. Overall, there is an increase in cell division and a modification in cellular self-degradation [17].

On the tissue level, this interaction could minimize inflammation by lowering prostaglandin E2, prostaglandin-endoperoxide synthase 2, interleukin 1 beta, tumor necrosis factor-alpha, cellular inflow of neutrophil granulocytes, oxidative stress, edema, and bleeding in a dose-dependent way. Also, PBM inhibits the pain receptors, which is assumed to be the cause of pain relief. PBM has been demonstrated to alleviate discomfort and encourage the shrinkage of erythema related to OLP lesions by utilizing wavelengths between 630 and 980 nm and output powers between 20 and 300 mW. Therefore, the objective of this clinical trial was to assess the effect of PBM using 980 nm diode laser in the management of erosive OLP in comparison to conventional corticosteroid therapy [18–21].

The research’s null hypothesis was that there wouldn’t be any statistically significant differences between the test group receiving PBM with 980 nm diode laser therapy for erosive OLP and the control group receiving conventional corticosteroids.

## Material and methods

### Study design

This study was performed as a two-arm randomized (1:1), controlled clinical trial. Forty-four patients suffering from symptomatic erosive oral lichen planus were randomly selected from the patient pool of outpatient clinic of the Oral Medicine, Periodontology, Diagnosis, and Radiology Department, Faculty of Dentistry, Alexandria University, Egypt, starting from June 2022 until March 2023.

The Research Ethics Committee at the Faculty of Dentistry Alexandria University approved the study (IRB No. 00010556- IORG 0008839- 0369-01/2022) in January 2022. The study was registered at the Clinical Trials Registry (NCT 05951361) on 14/7/2023. It also adhered to the criteria of the modified Helsinki code for human clinical investigations (2013) and CONSORT 2010 reporting guidelines for randomized clinical trials. Patients received a thorough explanation of the study protocol, and an informed consent was obtained from each patient [22, 23].

### Participants

Patients were enrolled in the study when they had erosive oral lichen planus diagnosed by clinical examination that complied with WHO modified criteria (2003) for OLP diagnosis, and the diagnosis was histopathologically confirmed [24]. All patients aged between 30 and 70 years old.

Patients were excluded when they were currently on corticosteroid therapy or had been on treatment during the past 3 months. Patients administering anti-inflammatory drugs, illicit drugs, any medications associated with oral lichenoid reactions, or treatment for cancer. Patients with any uncontrolled systemic diseases, pregnant or breast-feeding women, and smokers were all excluded. Also, the presence of amalgam restorations near the OLP lesions, dysplasia in histopathological examination, or presence of skin lesions were among the exclusion criteria [25–28].

### Sample size calculation

Rosner’s method was used to estimate the sample size. It was calculated by G-power 3.0.10. assuming study power = 80% and level of confidence = 95% based on Dillenburg et al., 2014 [29]. The mean (SD) reported pain scores measured by the visual analogue scale (VAS) after 3 months for the laser group was 0.79 (1.23) and for the steroid group was 2.81 (2.84). The sample size of 20 patients per group was found to be enough, however, it was initially increased to 22 patients per group in order to account for potential loss of follow-up [30, 31].

### Randomization, blinding, and allocation concealment

Participants were assigned to one of the two study groups using a computer-generated random allocation software utilising the permuted block technique.

The participant distribution was kept in opaque, sealed envelopes and opened after completing the oral examination and right before the application of the intervention [32]. The blinding of the patients and the main operator was difficult as the two groups had different treatment regimens. However, the statistician and the biochemist were blinded to the allocation of groups.

### Interventions

After taking the medical history, clinical examination, histopathological confirmation of the diagnosis, and before treatment, the following groups were randomly selected from the forty-four eligible patients:

- Group I (control group): received conventional therapy for erosive OLP lesions. Topical corticosteroids (0.1% triamcinolone acetonide preparation) were prescribed to the patients three times per day for 4 weeks in one direction, with no fluids or food allowed after the gel application for at least 1 h. Additionally, topical antifungal (2% miconazole oral gel) once daily for a duration of 4 weeks [33].
- Group II (photobiomodulation group): received photobiomodulation therapy by 980 nm diode laser and the treatment was continued up to 10 sessions for 5 weeks (2 sessions per week) by the same operator. The energy was distributed evenly over all of the mucosal lesions and the peri-lesional tissues up to 0.5 cm using a “spot” approach with a minor overlap. The probe was held perpendicularly in a non-contact mode at a distance of roughly 2 mm during each session, with 400 µm diameter fiber optic tip, and output power 300 mW. The power of the device was calibrated by the company’s technical support several times throughout the study period. In a continuous wave, the delivery time was around 4 s per point of application producing an energy of 1.2 J for each point. Depending on the size of the lesion being treated, different number of spots and different amount of energy were provided to the whole lesion. The used parameters were based on the research conducted by Cafaro et al., 2014 with modifications [27].

### Saliva sampling

Prior to saliva collection, all patients received step 1 and 2 therapy (oral hygiene recommendations, supra and subgingival scaling) one day before saliva collection in order to prevent blood contamination of saliva samples [34].

The samples of saliva were taken between 9 and 11 a.m. at baseline before treatment, 6 weeks, and 12 weeks postoperatively. Participants were informed to abstain teeth brushing, eating, or even drinking for at least two hours before collecting saliva.

Before saliva collection, they were instructed to cleanse their mouths using distilled water [35]. Afterwards, they were instructed to spit 5 ml of saliva in a dry, clean glass container for five minutes. Any saliva samples that were tinted with blood were immediately eliminated [36].

To eliminate cellular debris and bacteria, samples were immediately placed into dry, clean centrifuge tubes and centrifuged at 1,058 g for 5 min at 4 °C [37]. Following that, the supernatant was separated into minute aliquots and put in Eppendorf tubes with the patient’s sequential number on them.

To distinguish the baseline from the post-treatment samples, saliva samples were color coded. Before analysis, all saliva samples were kept at -80 °C [38]. The biochemical analysis was done at the Biochemistry Department of the Faculty of Medicine, Alexandria University, Egypt.

### Outcome measures

The following outcome measures were recorded at baseline, 6 weeks, and 12 weeks postoperatively.

#### Pain

Each patient was requested to rate their level of discomfort and agony (subjective clinical outcome) using a visual analogue scale (VAS), where score 0 means no pain and score 10 equal the worst pain ever felt [27].

#### Clinical size of the lesion

The lesion size was monitored according to the Thongprasom et al. [12] scoring system, where score 5 means erosive area with white striae > 1 cm^2^, score 4 means erosive area with white striae < 1 cm^2^, score 3 means presence of erythematous area > 1 cm^2^ with white striae, score 2 means erythematous area < 1 cm^2^ and white striae, score 1 means mild white striae only, while score 0 means total absence of lesions and only normal mucosa. Patients’ photographic records were recorded before treatment, 6 weeks, and 12 weeks postoperatively [27].

#### Salivary oxidative stress status (Marker of lipid peroxidation, Malondialdehyde (MDA))

The marker of lipid peroxidation (MDA) was measured using special kits (Biodiagnostic Diagnostic and Research Reagents, Cairo, Egypt).

The determination of MDA depended on the chemical reaction between MDA and thiobarbituric acid (TBA) in an acidic medium at 95 °C for 30 min, which ended in the formation of a reactive pink product of TBA. The absorbance of the reactive pink product is measured at 534 nm by Human Humalyzer Junior, GmbH. Germany [39].

### Statistical analysis

Version 20.0 of the IBM SPSS software program was utilized to investigate the data. The fundamental premise is that there is no difference in pain and lesion clinical scores between the two therapy approaches. Qualitative data was presented as percentages and numbers. However, to categorize quantitative data, the range (minimum and maximum), mean, standard deviation, median, and interquartile range (IQR) were utilized. Furthermore, the 5% level was used to establish the data’ significance. In order to analyze the two groups’ differences in terms of demographic information (gender, age), chi-square (× 2) and student t-tests (t-test) were utilized.

The non-parametric tests were used as the data was found to be not normally distributed after we checked it with Shapiro test. The variations among the two groups in terms of VAS, clinical score and salivary MDA levels were assessed using Mann Whitney test. Friedman test was used to analyze the changes in VAS, clinical score and salivary MDA levels over the course of each treatment group and Post Hoc test (Dunn’s) was used for additional analysis if there were any significant differences.

## Results

**Fig. 1:**
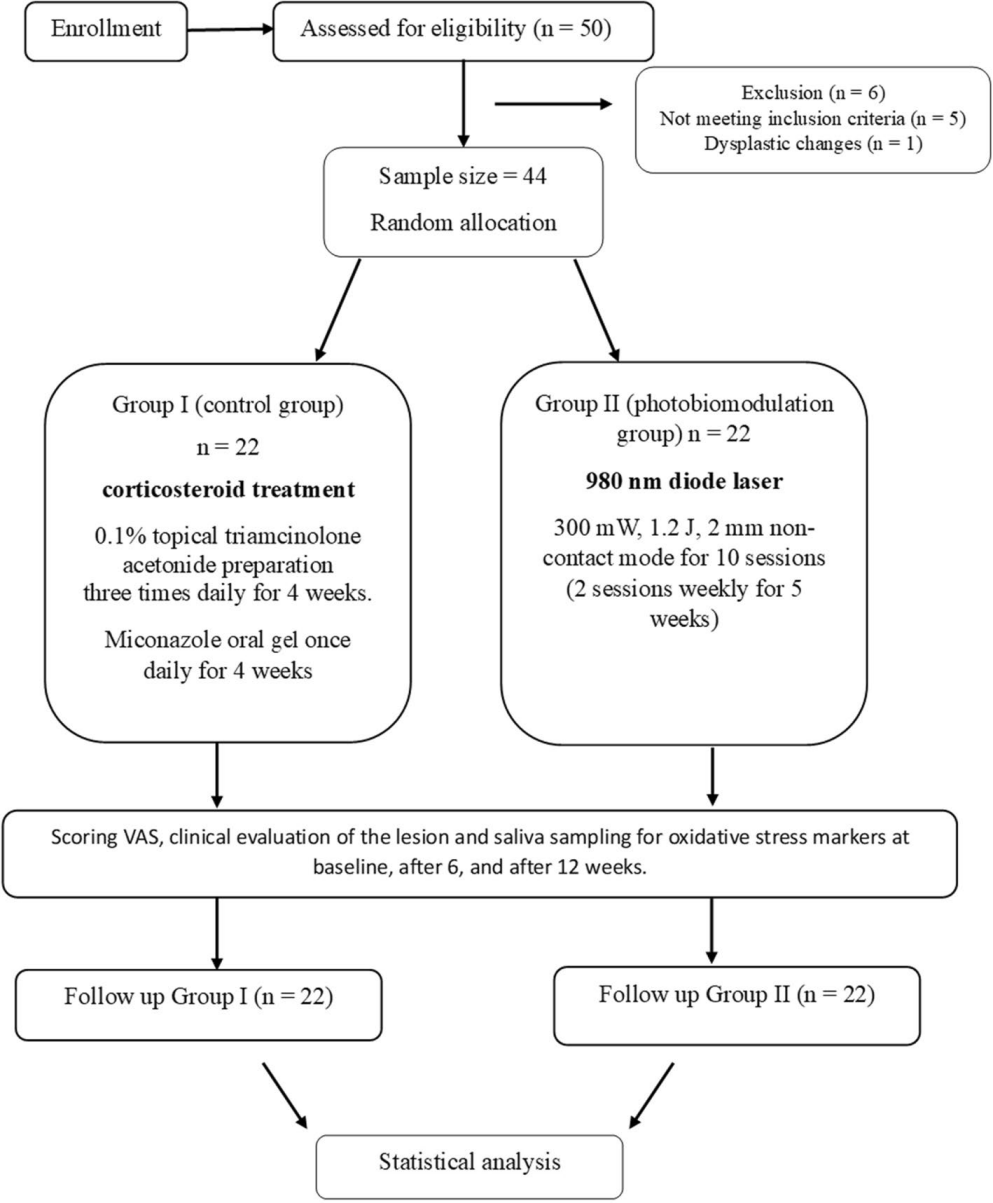
Showing the consort flow chart

A total of fifty patients were assessed for eligibility, but only forty-four patients fulfilled the inclusion criteria and were randomly selected for this randomized controlled clinical trial. All patients completed the study with a mean age mean ± SD 52.91 ± 12.41. No complications were experienced by the patients, as shown in Fig. 1 following the consort flow chart.

### Effect of treatment on Pain (VAS)

Data at baseline, 6 weeks, and 12 weeks postoperatively revealed a significant decrease in pain scores (VAS) within each group, with a mean ± SD of 7.14 ± 1.17 that decreased to 1.32 ± 0.84 (*p* < 0.001) for the laser group and 7.05 ± 0.84 diminished to 1.68 ± 0.65 (*p* < 0.001) for the control group, as shown in Table 1. There was no significant difference between the two groups regarding pain either at baseline or within the follow-up period (*p* = 0.468), as shown in Table 2.

**Table 1:** The changes in VAS between the three studied periods

VAS^a^	Baseline	After 6 Weeks	After 12 Weeks	Fr^b^	*p*
Photobiomodulation group (*n* = 22)					
Min. – Max	5.0 – 9.0	3.0 – 5.0	0.0 – 3.0	44.0	< 0.001
Mean ± SD^c^	7.14 ± 1.17	4.27 ± 0.77	1.32 ± 0.84		
Median (IQR)^d^	7.0(6.0 – 8.0)	4.0(4.0 – 5.0)	1.0(1.0 – 2.0)		
Significant between periods	*p*_1_ = 0.00, *p*_2_ < 0.001, *p*_3_ = 0.001^*^				
Control group (*n* = 22)					
Min. – Max	6.0 – 9.0	3.0 – 7.0	1.0 – 3.0	44.0	< 0.001
Mean ± SD	7.05 ± 0.84	4.64 ± 1.18	1.68 ± 0.65		
Median (IQR)	7.0(6.0 – 8.0)	4.0(4.0 – 5.0)	2.0(1.0 – 2.0)		
Significant between periods	*p*_1_ = 0.001, *p*_2_ < 0.001, *p*_3_ = 0.001				

p1: *p* value for comparing between Baseline and 6 Weeks

p2: *p* value for comparing between Baseline and 12 Weeks

p3: *p* value for comparing between 6 Weeks and After 12 Weeks

Statistically significant difference at *p* value ≤ 0.05

^*^p: *p* value for comparing between the studied groups

^a^Visual analogue scale

^b^Fr: Friedman test, significant difference between periods was done using Post Hoc Test (Dunn’s)

^c^*SD *Standard deviation

^d^*IQR *Inter quartile range

**Table 2:** The changes in VAS between two studied groups along the periods of the study

VAS	Photobiomodulation group (*n* = 22)	Control group (*n* = 22)	U^a^	p^*^
**Baseline**				
Min. – Max	5.0 – 9.0	6.0 – 9.0	227.00	0.712
Mean ± SD	7.14 ± 1.17	7.05 ± 0.84		
Median (IQR)	7.0(6.0 – 8.0)	7.0(6.0 – 8.0)		
**After 6 Weeks**				
Min. – Max	3.0 – 5.0	3.0 – 7.0	211.00	0.443
Mean ± SD	4.27 ± 0.77	4.64 ± 1.18		
Median (IQR)	4.0(4.0 – 5.0)	4.0(4.0 – 5.0)		
**After 12 Weeks**				
Min. – Max	0.0 – 3.0	1.0 – 3.0	178.50	0.105
Mean ± SD	1.32 ± 0.84	1.68 ± 0.65		
Median (IQR)	1.0(1.0 – 2.0)	2.0(1.0 – 2.0)		
**Decrease**	5.82 ± 1.68	5.36 ± 0.95	212.0	0.468

Statistically significant at *p* ≤ 0.05

^*^p *p* value for comparing between the studied groups

^a^U Mann Whitney test

### Effect of different treatments on clinical score

**Fig. 2:**
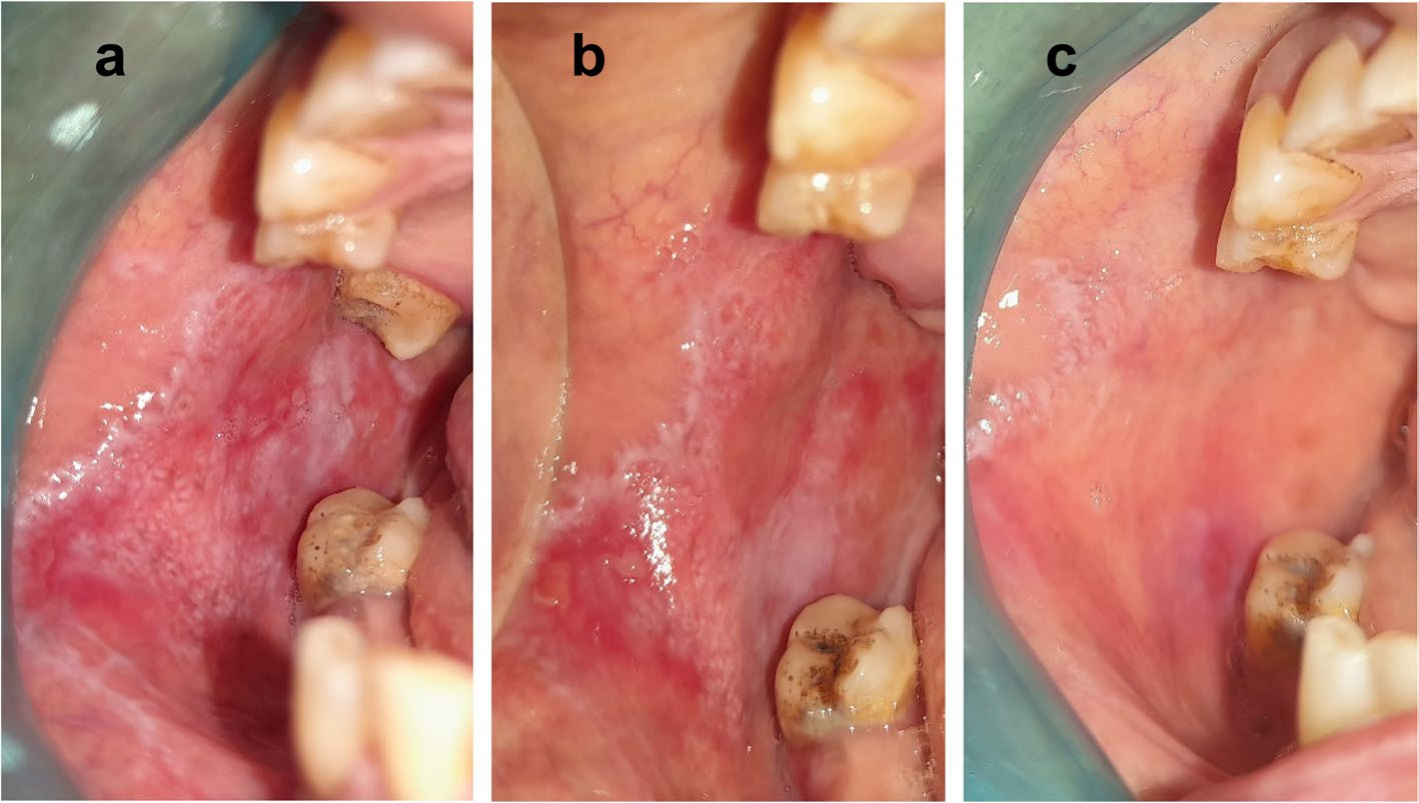
Effect of photobiomodulation therapy on erosive OLP case. **a** baseline. **b** 6 weeks. **c** 12 weeks

**Fig. 3:**
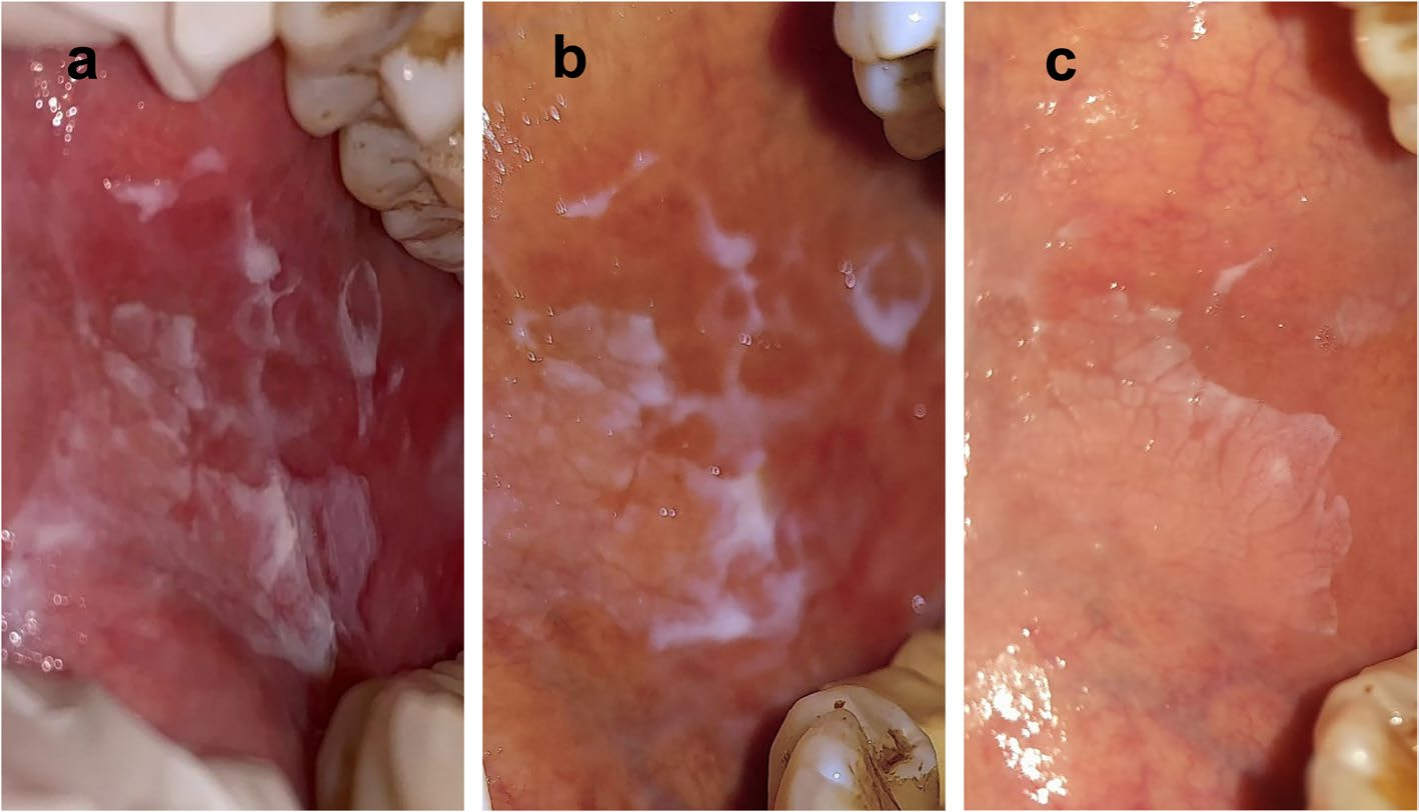
Effect of 0.1% topical triamcinolone therapy on erosive OLP case. **a** baseline. **b** 6 weeks. **c** 12 weeks

Concerning the clinical score of the lesions, scores dropped from 3.32 ± 0.99 to 0.77 ± 0.61 (*p* < 0.001) for the laser group and 3.23 ± 1.07 to 0.68 ± 0.72 (*p* < 0.001) for the control group, as shown in Table 3, indicating a statistically significant improvement within the same group from baseline until 12 weeks of follow-up, as shown in Figs. 2 and 3. However, there was no significant difference in clinical scores between the two groups (*p* = 0.821), as shown in Table 4.

**Table 3:** The changes in clinical scores of the lesion between the three studied periods

Clinical score	Baseline	After 6 Weeks	After 12 Weeks	**Fr**	***p***
Photobiomodulation group (*n* = 22)					
Min. – Max	2.0 – 5.0	1.0 – 3.0	0.0 – 2.0	43.070	< 0.001
Mean ± SD	3.32 ± 0.99	2.0 ± 0.69	0.77 ± 0.61		
Median (IQR)	3.0(3.0 – 4.0)	2.0(2.0 – 2.0)	1.0(0.0 – 1.0)		
Significant between periods	*p*_1_ = 0.001, *p*_2_ < 0.001, *p*_3_ = 0.003				
Control group (*n* = 22)					
Min. – Max	2.0 – 5.0	1.0 – 3.0	0.0 – 2.0	43.070	< 0.001
Mean ± SD	3.23 ± 1.07	1.95 ± 0.72	0.68 ± 0.72		
Median (IQR)	3.0(2.0 – 4.0)	2.0(1.0 – 2.0)	1.0(0.0 – 1.0)		
Significant between periods	*p*_1_ = 0.001, *p*_2_ < 0.001, *p*_3_ = 0.003

**Table 4:** Changes in clinical scores of the lesion between two studied groups along the study periods

Clinical score	Photobiomodulation group (*n* = 22)	Control group (*n* = 22)	U	*p*
**Baseline**				
Min. – Max	2.0 – 5.0	2.0 – 5.0	227.00	0.708
Mean ± SD	3.32 ± 0.99	3.23 ± 1.07		
Median (IQR)	3.0(3.0 – 4.0)	3.0(2.0 – 4.0)		
**After 6 Weeks**				
Min. – Max	1.0 – 3.0	1.0 – 3.0	233.50	0.827
Mean ± SD	2.0 ± 0.69	1.95 ± 0.72		
Median (IQR)	2.0(2.0 – 2.0)	2.0(1.0 – 2.0)		
**After 12 Weeks**				
Min. – Max	0.0 – 2.0	0.0 – 2.0	219.50	0.559
Mean ± SD	0.77 ± 0.61	0.68 ± 0.72		
Median (IQR)	1.0(0.0 – 1.0)	1.0(0.0 – 1.0)		
**Decrease**	2.55 ± 0.91	2.55 ± 1.06	233.00	0.821

### Effect of different treatments on salivary oxidative stress markers

The effect of various treatments on salivary oxidative stress markers, as shown in Table 5, represented a statistically significant reduction in salivary MDA levels within each group, with a mean ± SD of 7.33 ± 2.92 decreasing to 3.50 ± 2.20 (*p* < 0.001) for the laser group, and changing from 4.79 ± 1.70 to 1.61 ± 0.82 (*p* < 0.001) for the control group. According to Table 6, there was no statistically significant difference between the two groups’ MDA levels (*p* = 0.324).

**Table 5:** The changes in salivary MDA between the three studied periods

Salivary MDA^a^	Before	After 6 Weeks	After 12 Weeks	Fr	*p*
Photobiomodulation group (*n* = 22)					
Min. – Max	2.0 – 13.30	1.50 – 11.90	1.20 – 8.30	36.488	< 0.001
Mean ± SD	7.33 ± 2.92	5.98 ± 2.97	3.50 ± 2.20		
Median (IQR)	7.75(4.7 – 9.0)	6.0(3.5 – 7.9)	2.90(1.5 – 5.4)		
Significant between periods	*p*_1_ = 0.042, *p*_2_ < 0.001, *p*_3_ < 0.001				
Control group (*n* = 22)					
Min. – Max	1.10 – 7.30	0.60 – 6.0	0.50 – 3.40	32.345	< 0.001
Mean ± SD	4.79 ± 1.70	2.89 ± 1.83	1.61 ± 0.82		
Median (IQR)	4.90(4.1 – 6.0)	2.40(1.5 – 4.9)	1.45(0.90 – 2.1)		
Significant between periods	*p*_1_ = 0.003, *p*_2_ < 0.001, *p*_3_ = 0.007				

^a^Malondialdehyde

**Table 6:** The decrease in salivary MDA between two studied groups along the periods of the study

Salivary MDA	Photobiomodulation group (*n* = 22)	Control group (*n* = 22)	U	*p*
**Baseline**				
Min. – Max	2.0 – 13.30	1.10 – 7.30	105.50	0.001
Mean ± SD	7.33 ± 2.92	4.79 ± 1.70		
Median (IQR)	7.75(4.7 – 9.0)	4.90(4.1 – 6.0)		
**After 6 Weeks**				
Min. – Max	1.50 – 11.90	0.60 – 6.0	94.00	0.001
Mean ± SD	5.98 ± 2.97	2.89 ± 1.83		
Median (IQR)	6.0(3.5 – 7.9)	2.40(1.5 – 4.9)		
**After 12 Weeks**				
Min. – Max	1.20 – 8.30	0.50 – 3.40	101.00	0.001
Mean ± SD	3.50 ± 2.20	1.61 ± 0.82		
Median (IQR)	2.90(1.5 – 5.4)	1.45(0.90 – 2.1)		
**Decrease**	3.83 ± 2.17	3.18 ± 1.94	200.00	0.324

## Discussion

OLP is a chronic mucocutaneous condition for which long-term care is required to reduce symptoms as there is no known cure [6]. The first line of treatment for OLP has traditionally been topical and systemic corticosteroids; however, corticosteroids have been linked to severe side effects including mucosal thinning and oral candidiasis [40]. Therefore, alternative remedies were recommended.

Previous studies have shown that PBM has beneficial effects on a variety of inflammatory disorders, suggesting that this treatment may be an effective and promising therapy offering cellular metabolism enhancement and regenerative properties with a non-thermal impact on the living tissues. However, disagreement has emerged over its effect on OLP lesions as a result of varied PBM parameters employed and the significant risk of bias seen in earlier studies [41, 42]. Consequently, additional studies in this area were necessary, which was the initiative for conducting the present study.

In this randomized, parallel, controlled clinical trial, PBM showed similar effects to the gold standard therapy of corticosteroids in OLP patients, as both groups showed a significant improvement in pain and lesion clinical scores during the treatment period with no relapse observed during the follow-up period.

Similar effects were demonstrated in a study conducted by Cafaro et al., 2014, who managed unresponsive OLP lesions to topical steroids with a 980 nm diode laser [27]. In addition, Gambino et al., 2021, who compared the bio-stimulatory effect of 980 nm diode laser once weekly for eight weeks to 0.05% clobetasol propionate twice daily for 8 weeks, and reported a significant improvement of the oral mucosa and of the epithelium-connective interface for both groups [43].

Moreover, Mutafchieva et al., 2018 evaluated the effect of an 810 nm diode laser on long-standing erosive or atrophic OLP lesions [44]. Laser was applied three times weekly for a month. The authors found a significant reduction in pain as well as an improvement in the lesions’ clinical scores. Another study by Jajarm et al., 2011 showed that, using PBM with 630 nm diode laser twice weekly for a total of 10 sessions was as successful as the treatment utilizing dexamethasone mouth wash [45].

Furthermore, Dillenburg et al., 2014 and Ferri et al., 2020, reported that both the bio-modulation effects of 660 nm diode laser and topical steroid gel in managing erosive OLP patients demonstrated a significant reduction in pain with no difference among the two groups. Nevertheless, Ferri et al. noted that they achieved their results with fewer PBM sessions per week [25, 29].

On the other hand, some authors found that corticosteroids (dexamethasone and triamcinolone) improved OLP symptoms better than PBM, which is contradictory to all the aforementioned studies. El Shenawy et al., 2015 who used laser till tissue blenching and Othman et al., 2016 showed that topical corticosteroids were more effective than using a 970 nm diode laser in the treatment of OLP [26, 46]. In addition, Kazancioglu and Erisen, 2015 reported the higher impact of ozone and corticosteroid therapies than 880-nm bio-stimulating effect [47]. The difference in the results of these studies compared to the current study may be owed to the use of excessively high laser power output (2000–3000 mW).

Ferri et al., 2020 stated that, thermal effects caused by high power output in PBM may contribute to the lack of effectiveness of the therapy [25]. Moreover, Caruso-Davis et al., 2011 considered the laser output power for PBM is essential to be below 500 mW (depending on the target tissue) without raising the temperature; otherwise, PBM tends to have no significant effect. The same concept was emphasized by de Ferities et al., 2015 who stated that, improper parameters (too high energy) can result in ineffective treatment. According to this study, the biphasic dose response curve, also referred to as hormesis, demonstrated that inadequate or excessive doses can result in insignificant effects or unwarranted inhibition.

This explanation is supported by “Arndt-Schulz Law”. According to this law, weak stimuli marginally boost vital activity, while stronger stimuli enhance it until a maximum level is attained. However, excessively strong stimuli stifle it, leading to a negative response [48]. This may explain the ineffectiveness of PBM in the previous studies conducted by Kazancioglu et al., and Othman et al. [26, 47]. Moreover, the laser dose within the PBM therapeutic window should not cause any observable tissue changes, this may explain the ineffectiveness of laser therapy in the study of El Shenawy et al. who used high dosage until tissue blanching [46, 48].

In our study, in comparison to baseline, the lesions showed a significant reduction in clinical size with a concurrent increase in reticular OLP lesions and healing of erosive lesions at 6 weeks and further improvement at 12 weeks of follow-up, as evaluated by Thongprasom et al. (2003) scoring system, with no difference between the two groups, showing that both therapies enhanced an improvement in the clinical appearance of OLP [12].

Thongprasom’s score was selected because it is relatively simple and easily reproducible scoring system which has been frequently utilized in many OLP studies (Arduino et al., 2018; Cosgarea et al., 2020; Thongprasom et al., 1992) allowing the comparison of the results obtained from the current study to others [49–51]. However, none of the scoring systems available to date could exactly reflect the changes in the lesions and the improvement of the patient’s condition [52].

In our study, we assessed the effect of PBM on erosive OLP management by measuring salivary MDA levels, which are the most commonly studied byproduct of lipid peroxidation and have been regarded as a valid biomarker for the levels of oxidative stress [53].

Oxidative stress markers can be measured in various human specimens and tissues, such as saliva, serum, urine, and tissue homogenate [54]. The decision to use salivary samples in this clinical trial as a diagnostic tool was based upon various benefits, they offer over blood samples. Collecting saliva is non-invasive, rapid and affordable diagnostic method that has been proven to be efficient in diagnosis of both systemic and oral disorders. Moreover, saliva is the body’s primary defense against oxidative stress and regarded as a mirror for the entire body as it contains serum components [55–57]. The decision to use salivary samples in this clinical trial as a diagnostic tool was based upon the various benefits, they offer over blood samples. Collecting saliva is a non-invasive, rapid, and affordable diagnostic method that has been proven to be efficient in the diagnosis of both systemic and oral disorders. Moreover, saliva is the body’s primary defence against oxidative stress and is regarded as a mirror for the entire body as it contains serum components.

In this study, salivary MDA levels decreased significantly in response to treatment in the corticosteroid group and PBM group as measured at baseline, 6 and 12 weeks after treatment, with no significant difference between groups.

These results can be interpreted by the fact that, PBM increases ATP production and elevates the cellular oxygen level, which may lead to scaling down ROS and MDA [58]. Whereas corticosteroids are known for their anti-inflammatory and immunosuppressive effects, which can decrease the production of ROS and lipid peroxidation products MDA, Sanner et al., 2002 concluded that corticosteroids directly suppress the generation of intracellular reactive oxygen species, this impact may play a role in the anti-inflammatory effects of these substances [59]. Tsai CC et al., 2007 stated that glucocorticoids are associated with a decrease in the marker of oxidative stress [60].

Many studies have found that OLP patients have higher serum and salivary MDA levels than healthy individuals [61]. However, only a small number of studies have examined the levels of MDA in response to treatment modalities for oral diseases [62].

Qataya et al., 2020 studied the levels of salivary MDA in response to the administration of topical corticosteroids and topical and systemic selenium in OLP patients. They found that salivary MDA levels significantly decreased in patients who received topical corticosteroids and systemic selenium, while patients receiving topical selenium showed a statistically non-significant decrease. The difference between the results of our study and the group of topical selenium in the study by Qataya et al. could be explained by the fact that topical selenium might lack a prominent effect on oxidative stress in OLP lesions [63]. 

In line with our results, a study conducted by Rai et al., 2010 showed a significant reduction in salivary and serum MDA levels and other oxidative markers in managing oral potentially malignant lesions treated with curcumin [37].

Conversely, participants who received corticosteroid therapy for 2 weeks in a study conducted by Hashemy et al., 2016 exhibited no significant correlation in serum MDA levels compared to the control group. The contradiction between the results of this study and the study by Hashemy et al. could be attributed to the positive effects of PBM on oxidative stress in the laser group. In the corticosteroid group, the decrease in salivary MDA could be due to the longer duration of corticoid therapy administration (4 weeks) compared to the study by Hashemy et al., 2016 [62].

In spite of the promising results obtained from the present study, some limitations were inevitable. The main limitations were the small sample size and the short follow-up period. As well, this clinical trial did not include healthy controls to assess salivary levels of MDA in age and gender-matched healthy individuals.

Further studies with a larger sample size and longer follow-up periods are recommended to optimize the most suitable parameters of PBM for erosive oral lichen planus lesions and to confirm the validity of MDA as a biomarker for the disease progression and the effectiveness of the applicable treatment.

## Conclusions

In conclusion, PBM demonstrates a safe therapy with no apparent side effects. It was as effective as the use of topical 0.1% triamcinolone acetonide therapy for the treatment of EOLP lesions, omitting the adverse effects of the corticosteroid therapy.

Salivary MDA could be used as an oxidative stress biomarker to monitor OLP severity and its response to different treatment modalities.

## Data Availability

The data that support the findings of this study are available from the corresponding author upon reasonable request.

## Abbreviations

OLP: Oral lichen planus
PBM: Photobiomodulation
MDA: Malondialdehyde
SD: Standard deviation
OS: Oxidative stress
ROS: Reactive oxygen species
TNF-α: Tumor necrosis factor alpha
MMP: Matrix metalloproteinase
ATP: Adenosine triphosphate
CCCO: Cytochrome C oxidase
NO: Nitric oxide
RNA: Ribonucleic acid
DNA: Deoxyribonucleic acid
NCT: National Clinical Trial number
WHO: World Health Organization
VAS: Visual Analogue Scale
TBA: Thiobarbituric acid
SPSS: Statistical Package for the Social Sciences
x2: Chi-square test
t-test: Student t-tests
U test: Mann Whitney test
LLLT: Low level laser therapy
EOLP: Erosive Oral lichen planus
